# Supplementary material for: Exploring Applications of Artificial Intelligence in Critical Care Nursing: A Systematic Review
Source: Nurs Rep. 2025 Feb 4;15(2):55. doi: 10.3390/nursrep15020055 (PMC11857867; doi:10.3390/nursrep15020055)
Supplement: Supplementary file 1 [file nursrep-15-00055-s001.zip › nursrep-3423194-supplementary.pdf]

Supplementary Table S1. The PRISMA 2020 statement.

Section and Topic	Item #	Checklist item	Location where item is reported
**TITLE**			
Title	1	Identify the report as a systematic review.	Title
**ABSTRACT**			
Abstract	2	See the PRISMA 2020 for Abstracts checklist.	Abstract
**INTRODUCTION**			
Rationale	3	Describe the rationale for the review in the context of existing knowledge.	1. Introduction
Objectives	4	Provide an explicit statement of the objective(s) or question(s) the review addresses.	1. Introduction
**METHODS**			
Eligibility criteria	5	Specify the inclusion and exclusion criteria for the review and how studies were grouped for the syntheses.	2.2 Inclusion and exclusion criteria
Information sources	6	Specify all databases, registers, websites, organisations, reference lists and other sources searched or consulted to identify studies. Specify the date when each source was last searched or consulted.	2.1 Study design
Search strategy	7	Present the full search strategies for all databases, registers and websites, including any filters and limits used.	Table S1: Search strategy
Selection process	8	Specify the methods used to decide whether a study met the inclusion criteria of the review, including how many reviewers screened each record and each report retrieved, whether they worked independently, and if applicable, details of automation tools used in the process.	2.3 Study selection
Data collection process	9	Specify the methods used to collect data from reports, including how many reviewers collected data from each report, whether they worked independently, any processes for obtaining or confirming data from study investigators, and if applicable, details of automation tools used in the process.	2.4 Data extraction
Data items	10a	List and define all outcomes for which data were sought. Specify whether all results that were compatible with each outcome domain in each study were sought (e.g. for all measures, time points, analyses), and if not, the methods used to decide which results to collect.	2.2 Inclusion and exclusion criteria<br>2.3 Study selection
	10b	List and define all other variables for which data were sought (e.g. participant and intervention characteristics, funding sources). Describe any assumptions made about any missing or unclear information.	Not performed
Study risk of bias assessment	11	Specify the methods used to assess risk of bias in the included studies, including details of the tool(s) used, how many reviewers assessed each study and whether they worked independently, and if applicable, details of automation tools used in the process.	2.5 Studies' quality assessment
Effect measures	12	Specify for each outcome the effect measure(s) (e.g. risk ratio, mean difference) used in the synthesis or presentation of results.	Not performed
Synthesis methods	13a	Describe the processes used to decide which studies were eligible for each synthesis (e.g. tabulating the study intervention characteristics and comparing against the planned groups for each synthesis (item #5)).	2.2 Inclusion and exclusion criteria<br>2.3 Study selection
	13b	Describe any methods required to prepare the data for presentation or synthesis, such as handling of missing summary statistics, or data conversions.	2.4 Data extraction
	13c	Describe any methods used to tabulate or visually display results of individual studies and syntheses.	2.6 Knowledge synthesis
	13d	Describe any methods used to synthesize results and provide a rationale for the choice(s). If meta-analysis was performed, describe the model(s), method(s) to identify the presence and extent of statistical heterogeneity, and software package(s) used.	2.6 Knowledge synthesis
	13e	Describe any methods used to explore possible causes of heterogeneity among study results (e.g. subgroup analysis, meta-regression).	Not performed

Supplementary Table S1. The PRISMA 2020 statement.

Section and Topic	Item #	Checklist item	Location where item is reported
	13f	Describe any sensitivity analyses conducted to assess robustness of the synthesized results.	Not performed
Reporting bias assessment	14	Describe any methods used to assess risk of bias due to missing results in a synthesis (arising from reporting biases).	Not performed
Certainty assessment	15	Describe any methods used to assess certainty (or confidence) in the body of evidence for an outcome.	Not performed
**RESULTS**			
Study selection	16a	Describe the results of the search and selection process, from the number of records identified in the search to the number of studies included in the review, ideally using a flow diagram.	Figure 1. PRISMA diagram of study selection and inclusion.<br>3.1 General Characteristics
	16b	Cite studies that might appear to meet the inclusion criteria, but which were excluded, and explain why they were excluded.	Figure 1. PRISMA diagram of study selection and inclusion.
Study characteristics	17	Cite each included study and present its characteristics.	Table 1. General characteristics of the included studies.
Risk of bias in studies	18	Present assessments of risk of bias for each included study.	Table S2: Quality appraisal of studies included
Results of individual studies	19	For all outcomes, present, for each study: (a) summary statistics for each group (where appropriate) and (b) an effect estimate and its precision (e.g. confidence/credible interval), ideally using structured tables or plots.	Table 3. Training models' techniques, model of AI and performance measures
Results of syntheses	20a	For each synthesis, briefly summarise the characteristics and risk of bias among contributing studies.	Table S2: Quality appraisal of studies included
	20b	Present results of all statistical syntheses conducted. If meta-analysis was done, present for each the summary estimate and its precision (e.g. confidence/credible interval) and measures of statistical heterogeneity. If comparing groups, describe the direction of the effect.	3.1 Training models' techniques<br>3.2 Type of data approach
	20c	Present results of all investigations of possible causes of heterogeneity among study results.	3.1 Training models' techniques<br>3.2 Type of data approach
	20d	Present results of all sensitivity analyses conducted to assess the robustness of the synthesized results.	
Reporting biases	21	Present assessments of risk of bias due to missing results (arising from reporting biases) for each synthesis assessed.	Not reported
Certainty of evidence	22	Present assessments of certainty (or confidence) in the body of evidence for each outcome assessed.	Not reported
**DISCUSSION**			
Discussion	23a	Provide a general interpretation of the results in the context of other evidence.	4. Discussion
	23b	Discuss any limitations of the evidence included in the review.	4. Discussion
	23c	Discuss any limitations of the review processes used.	4.2 Strengths and limitations
	23d	Discuss implications of the results for practice, policy, and future research.	4.1 Implications for Nursing Practice
**OTHER INFORMATION**			
Registration and protocol	24a	Provide registration information for the review, including register name and registration number, or state that the review was not registered.	2.1 Study design<br>Declarations at the end of the manuscript

Supplementary Table S1. The PRISMA 2020 statement.

Section and Topic	Item #	Checklist item	Location where item is reported
	24b	Indicate where the review protocol can be accessed, or state that a protocol was not prepared.	2.1 Study design
	24c	Describe and explain any amendments to information provided at registration or in the protocol.	Not performed
Support	25	Describe sources of financial or non-financial support for the review, and the role of the funders or sponsors in the review.	Declarations at the end of the manuscript
Competing interests	26	Declare any competing interests of review authors.	Declarations at the end of the manuscript
Availability of data, code and other materials	27	Report which of the following are publicly available and where they can be found: template data collection forms; data extracted from included studies; data used for all analyses; analytic code; any other materials used in the review.	Declarations at the end of the manuscript

From: Page MJ, McKenzie JE, Bossuyt PM, Boutron I, Hoffmann TC, Mulrow CD, et al. The PRISMA 2020 statement: an updated guideline for reporting systematic reviews. BMJ 2021;372:n71. doi: 10.1136/bmj.n71. This work is licensed under CC BY 4.0. To view a copy of this license, visit https://creativecommons.org/licenses/by/4.0/

Supplementary Table S2. Search strategy.

DATABASE	SEARCH STRING
SCOPUS	TITLE-ABS-KEY ("Critical Care" OR "Intensive Care" OR "Surgical Intensive Care" OR "ICU" OR "Critical Illness" OR "Critically Ill" OR "Intensive care medicine" OR "Recovery Room" OR "Operating Room" OR "Operating theatre" OR "Postoperative Care" OR "Subacute Care" OR "Sub-Acute Care" OR "Sub Acute Care" OR "Postacute Care" OR "Post-acute Care" OR "Post acute Care" OR "Emergency Medical Services" OR "Medical Emergency Service" OR "Prehospital Emergency Care" OR "Prehospital Emergency" OR "Prehospital Care" OR "Emergicenter" OR "Emergency Care" OR "Emergency Health Service" OR "Hospital Emergency Services" OR "Emergency Hospital Service" OR "Hospital Emergency Room" OR "Accident and Emergency Department" OR "Emergency Ward" OR "Emergency Department" OR "Emergency Unit" OR "Hospital Emergency Service" OR "Emergency Room" OR "Hospital Emergency Services Utilization" OR "Emergency Services Utilization" OR "Emergency Outpatient Unit" OR "Trauma Center" OR "Ambulance" OR "Emergency Mobile Unit" OR "Mobile Emergency Unit" OR "Acute Care " OR "Acute Illness" OR "Acute Disease" OR "Intensive Treatment Unit")<br>AND TITLE-ABS-KEY ("Artificial Intelligence" OR "Computational Intelligence" OR "Machine Intelligence" OR "Computer Vision" OR "Knowledge Acquisition Computer" OR "Knowledge Representation computer" OR "Knowledge Representations Computer" OR "Deep learning" OR "Hierarchical Learning" OR "Machine learning" OR "Transfer Learning" OR "Data Mining" OR "Text Mining" OR "Supervised Machine Learning" OR "Supervised Learning" OR "Semi-supervised Learning" OR "Semi supervised Learning" OR "Learning from Labeled Data" OR "Unsupervised Machine Learning" OR "Unsupervised Learning" OR "artificial neural network" OR "Neural Network" OR "Natural Language Processing" OR "chat-gpt" OR "chatgpt" OR "gpt")<br>AND TITLE-ABS-KEY (“Nursing” OR “Critical Care Nursing” OR “Intensive Care Nursing” OR “Emergency Nursing” OR “Emergency Room Nursing” OR “Perioperative Nursing” OR “Operating Room Nursing” OR “Postanesthesia Nursing” OR “Post-Anesthesia Nursing” OR “Post Anesthesia Nursing” OR “Post Surgical Nursing” OR “Post-Surgical Nursing” OR “Recovery Room Nursing” OR “Trauma Nursing” OR “Nurse”)
WEB OF SCIENCE	("Critical Care" OR "Intensive Care" OR "Surgical Intensive Care" OR "ICU" OR "Critical Illness" OR "Critically Ill" OR "Intensive care medicine" OR "Recovery Room" OR "Operating Room" OR "Operating theatre" OR "Postoperative Care" OR "Subacute Care" OR "Sub-Acute Care" OR "Sub Acute Care" OR "Postacute Care" OR "Post-acute Care" OR "Post acute Care" OR "Emergency Medical Services" OR "Medical Emergency Service" OR "Prehospital Emergency Care" OR "Prehospital Emergency" OR "Prehospital Care" OR "emergicenters" OR "Emergency Care" OR "Emergency Health Service" OR "Hospital Emergency Services" OR "Emergency Hospital Service" OR "Hospital Emergency Room" OR "Accident and Emergency Department" OR "Emergency Ward" OR "Emergency Department" OR "Emergency Unit" OR "Hospital Emergency Service" OR "Emergency Room" OR "Hospital Emergency Services Utilization" OR "Emergency Services Utilization" OR "Emergency Outpatient Unit" OR "Trauma Center" OR "Ambulance" OR

	"Emergency Mobile Unit" OR "Mobile Emergency Unit" OR "Acute Care " OR "Acute Illness" OR "Acute Disease" OR "Intensive Treatment Unit") (Title) and ("Artificial Intelligence" OR "Computational Intelligence" OR "Machine Intelligence" OR "Computer Vision" OR "Knowledge Acquisition Computer" OR "Knowledge Representation computer" OR "Knowledge Representations Computer" OR "Deep learning" OR "Hierarchical Learning" OR "Machine learning" OR "Transfer Learning" OR "Data Mining" OR "Text Mining" OR "Supervised Machine Learning" OR "Supervised Learning" OR "Semi-supervised Learning" OR "Semi supervised Learning" OR "Learning from Labeled Data" OR "Unsupervised Machine Learning" OR "Unsupervised Learning" OR "artificial neural network" OR "Neural Network" OR "Natural Language Processing" OR "chat-gpt" OR "chatgpt" OR "gpt") (Title) and ("Nursing" OR "Critical Care Nursing" OR "Intensive Care Nursing" OR "Emergency Nursing" OR "Emergency Room Nursing" OR "Perioperative Nursing" OR "Operating Room Nursing" OR "Postanesthesia Nursing" OR "Post-Anesthesia Nursing" OR "Post Anesthesia Nursing" OR "Post Surgical Nursing" OR "Post-Surgical Nursing" OR "Recovery Room Nursing" OR "Trauma Nursing" OR "Nurse") (Title) or ("Critical Care" OR "Intensive Care" OR "Surgical Intensive Care" OR "ICU" OR "Critical Illness" OR "Critically Ill" OR "Intensive care medicine" OR "Recovery Room" OR "Operating Room" OR "Operating theatre" OR "Postoperative Care" OR "Subacute Care" OR "Sub-Acute Care" OR "Sub Acute Care" OR "Postacute Care" OR "Post-acute Care" OR "Post acute Care" OR "Emergency Medical Services" OR "Medical Emergency Service" OR "Prehospital Emergency Care" OR "Prehospital Emergency" OR "Prehospital Care" OR "emergicenters" OR "Emergency Care" OR "Emergency Health Service" OR "Hospital Emergency Services" OR "Emergency Hospital Service" OR "Hospital Emergency Room" OR "Accident and Emergency Department" OR "Emergency Ward" OR "Emergency Department" OR "Emergency Unit" OR "Hospital Emergency Service" OR "Emergency Room" OR "Hospital Emergency Services Utilization" OR "Emergency Services Utilization" OR "Emergency Outpatient Unit" OR "Trauma Center" OR "Ambulance" OR "Emergency Mobile Unit" OR "Mobile Emergency Unit" OR "Acute Care " OR "Acute Illness" OR "Acute Disease" OR "Intensive Treatment Unit") (Abstract) and ("Artificial Intelligence" OR "Computational Intelligence" OR "Machine Intelligence" OR "Computer Vision" OR "Knowledge Acquisition Computer" OR "Knowledge Representation computer" OR "Knowledge Representations Computer" OR "Deep learning" OR "Hierarchical Learning" OR "Machine learning" OR "Transfer Learning" OR "Data Mining" OR "Text Mining" OR "Supervised Machine Learning" OR "Supervised Learning" OR "Semi-supervised Learning" OR "Semi supervised Learning" OR "Learning from Labeled Data" OR "Unsupervised Machine Learning" OR "Unsupervised Learning" OR "artificial neural network" OR "Neural Network" OR "Natural Language Processing" OR "chat-gpt" OR "chatgpt" OR "gpt") (Abstract) and ("Nursing" OR "Critical Care Nursing" OR "Intensive Care Nursing" OR "Emergency Nursing" OR "Emergency Room Nursing" OR "Perioperative Nursing" OR "Operating Room Nursing" OR "Postanesthesia Nursing" OR "Post-Anesthesia Nursing" OR "Post Anesthesia Nursing" OR "Post Surgical Nursing" OR "Post-Surgical Nursing" OR "Recovery Room Nursing" OR "Trauma Nursing" OR "Nurse") (Abstract)

CINAHL	(TI ("Critical Care" OR "Intensive Care" OR "Surgical Intensive Care" OR "ICU" OR "Critical Illness" OR "Critically Ill" OR "Intensive care medicine" OR "Recovery Room" OR "Operating Room" OR "Operating theatre" OR "Postoperative Care" OR "Subacute Care" OR "Sub-Acute Care" OR "Sub Acute Care" OR "Postacute Care" OR "Post-acute Care" OR "Post acute Care" OR "Emergency Medical Services" OR "Medical Emergency Service" OR "Prehospital Emergency Care" OR "Prehospital Emergency" OR "Prehospital Care" OR "Emergicenter" OR "Emergency Care" OR "Emergency Health Service" OR "Hospital Emergency Services" OR "Emergency Hospital Service" OR "Hospital Emergency Room" OR "Accident and Emergency Department" OR "Emergency Ward" OR "Emergency Department" OR "Emergency Unit" OR "Hospital Emergency Service" OR "Emergency Room" OR "Hospital Emergency Services Utilization" OR "Emergency Services Utilization" OR "Emergency Outpatient Unit" OR "Trauma Center" OR "Ambulance" OR "Emergency Mobile Unit" OR "Mobile Emergency Unit" OR "Acute Care " OR "Acute Illness" OR "Acute Disease" OR "Intensive Treatment Unit") OR MH ("Critical Care" OR "Operating Rooms" OR "Postoperative Care" OR "Subacute Care" OR "Emergency Medical Services")) AND (TI (“Artificial Intelligence" OR "Computational Intelligence" OR "Machine Intelligence" OR "Computer Vision" OR "Knowledge Acquisition Computer" OR "Knowledge Representation computer" OR "Knowledge Representations Computer" OR "Deep learning" OR "Hierarchical Learning" OR "Machine learning" OR "Transfer Learning" OR "Data Mining" OR "Text Mining" OR "Supervised Machine Learning" OR "Supervised Learning" OR "Semi-supervised Learning" OR "Semi supervised Learning" OR "Learning from Labeled Data" OR "Unsupervised Machine Learning" OR "Unsupervised Learning" OR "artificial neural network" OR "Neural Network" OR "Natural Language Processing" OR "chat-gpt" OR "chatgpt" OR "gpt") OR MH ("Artificial Intelligence" OR "Deep Learning" OR "Machine Learning" OR "Data Mining" OR "Natural Language Processing" OR "Neural Networks (Computer)")) AND (TI (“Nursing” OR “Critical Care Nursing” OR “Intensive Care Nursing” OR “Emergency Nursing” OR “Emergency Room Nursing” OR “Perioperative Nursing” OR “Operating Room Nursing” OR “Postanesthesia Nursing” OR “Post-Anesthesia Nursing” OR “Post Anesthesia Nursing” OR “Post Surgical Nursing” OR “Post-Surgical Nursing” OR “Recovery Room Nursing” OR “Trauma Nursing” OR “Nurse”) OR MH ("Nursing" OR "Critical care Nursing" OR "Emergency Nursing" OR "Perioperative Nusing" OR "Trauma Nursing" OR “Nurses”)) OR (AB ("Critical Care" OR "Intensive Care" OR "Surgical Intensive Care" OR "ICU" OR "Critical Illness" OR "Critically Ill" OR "Intensive care medicine" OR "Recovery Room" OR "Operating Room" OR "Operating theatre" OR "Postoperative Care" OR "Subacute Care" OR "Sub-Acute Care" OR "Sub Acute Care" OR "Postacute Care" OR "Post-acute Care" OR "Post acute Care" OR "Emergency Medical Services" OR "Medical Emergency Service" OR "Prehospital Emergency Care" OR "Prehospital Emergency" OR "Prehospital Care" OR "Emergicenter" OR "Emergency Care" OR "Emergency Health Service" OR "Hospital Emergency Services" OR "Emergency Hospital Service" OR "Hospital Emergency Room" OR "Accident and Emergency Department" OR "Emergency Ward" OR "Emergency Department" OR "Emergency Unit" OR "Hospital Emergency Service" OR "Emergency Room" OR "Hospital Emergency Services Utilization" OR "Emergency Services Utilization" OR

	"Emergency Outpatient Unit" OR "Trauma Center" OR "Ambulance" OR "Emergency Mobile Unit" OR "Mobile Emergency Unit" OR "Acute Care " OR "Acute Illness" OR "Acute Disease" OR "Intensive Treatment Unit") OR MH ("Critical Care" OR "Operating Rooms" OR "Postoperative Care" OR "Subacute Care" OR "Emergency Medical Services")) AND (AB ("Artificial Intelligence" OR "Computational Intelligence" OR "Machine Intelligence" OR "Computer Vision" OR "Knowledge Acquisition Computer" OR "Knowledge Representation computer" OR "Knowledge Representations Computer" OR "Deep learning" OR "Hierarchical Learning" OR "Machine learning" OR "Transfer Learning" OR "Data Mining" OR "Text Mining" OR "Supervised Machine Learning" OR "Supervised Learning" OR "Semi-supervised Learning" OR "Semi supervised Learning" OR "Learning from Labeled Data" OR "Unsupervised Machine Learning" OR "Unsupervised Learning" OR "artificial neural network" OR "Neural Network" OR "Natural Language Processing" OR "chat-gpt" OR "chatgpt" OR "gpt") OR MH ("Artificial Intelligence" OR "Deep Learning" OR "Machine Learning" OR "Data Mining" OR "Natural Language Processing" OR "Neural Networks (Computer)")) AND (AB ("Nursing" OR "Critical Care Nursing" OR "Intensive Care Nursing" OR "Emergency Nursing" OR "Emergency Room Nursing" OR "Perioperative Nursing" OR "Operating Room Nursing" OR "Postanesthesia Nursing" OR "Post-Anesthesia Nursing" OR "Post Anesthesia Nursing" OR "Post Surgical Nursing" OR "Post-Surgical Nursing" OR "Recovery Room Nursing" OR "Trauma Nursing" OR "Nurse") OR MH ("Nursing" OR "Critical care Nursing" OR "Emergency Nursing" OR "Perioperative Nusing" OR "Trauma Nursing" OR "Nurses"))
PUBMED	("Critical Care"[Mesh] OR "Critical Care" [Title/Abstract] OR "Intensive Care" [Title/Abstract] OR "Surgical Intensive Care" [Title/Abstract]OR "ICU" [Title/Abstract] OR "Critical Illness" [Title/Abstract] OR "Critically Ill" [Title/Abstract] OR "Intensive care medicine" [Title/Abstract] OR "Recovery Room" [Title/Abstract] OR "Operating Room" [Mesh] OR "Operating Room" [Title/Abstract] OR "Operating theatre" [Title/Abstract] OR "Postoperative Care"[Mesh] OR "Postoperative Care" [Title/Abstract] OR "Subacute Care"[Mesh] OR "Sub-Acute Care" [Title/Abstract] OR "Sub Acute Care" [Title/Abstract] OR "Postacute Care" [Title/Abstract] OR "Post-acute Care" [Title/Abstract] OR "Post acute Care" [Title/Abstract] OR "Emergency Medical Services"[Mesh] OR "Medical Emergency Service" [Title/Abstract] OR "Emergency Medical Service" [Title/Abstract] OR "Prehospital Emergency Care" [Title/Abstract] OR "Prehospital Emergency" [Title/Abstract] OR "Prehospital Care" [Title/Abstract] OR "Emergicenter"[Title/Abstract] OR "Emergency Care"[Title/Abstract] OR "Emergency Health Service"[Title/Abstract] OR "Emergency Service, Hospital"[Mesh] OR "Hospital Emergency Service"[Title/Abstract] OR "Emergency Hospital Service"[Title/Abstract] OR "Hospital Emergency Room"[Title/Abstract] OR "Accident and Emergency Department"[Title/Abstract] OR "Emergency Ward"[Title/Abstract] OR "Emergency Department"[Title/Abstract] OR "Emergency Unit"[Title/Abstract] OR "Hospital Emergency Service"[Title/Abstract] OR "Emergency Room"[Title/Abstract] OR "Hospital Emergency Services Utilization"[Title/Abstract] OR "Emergency Services Utilization"[Title/Abstract] OR "Emergency Outpatient Unit"[Title/Abstract] OR "Trauma Center"[Title/Abstract] OR "Ambulance"[Title/Abstract] OR "Emergency Mobile Unit"[Title/Abstract] OR

	“Mobile Emergency Unit”[Title/Abstract] OR “Acute Care ”[Title/Abstract] OR “Acute Illness”[Title/Abstract] OR “Acute Disease”[Title/Abstract] OR “Intensive Treatment Unit”[Title/Abstract]) AND ("Artificial Intelligence"[Mesh] OR "Artificial Intelligence"[Title/Abstract] OR "Computational Intelligence"[Title/Abstract] OR "Machine Intelligence"[Title/Abstract] OR "Computer Vision"[Title/Abstract] OR “Knowledge Acquisition Computer”[Title/Abstract] OR “Knowledge Representation computer”[Title/Abstract] OR “Knowledge Representations Computer”[Title/Abstract] OR "Deep Learning"[Mesh] OR “Deep learning”[Title/Abstract] OR “Hierarchical Learning”[Title/Abstract] OR “Machine learning”[Mesh] OR “Machine learning”[Title/Abstract] OR “Transfer Learning”[Title/Abstract] OR "Data Mining"[Mesh] OR “Data Mining”[Title/Abstract] OR “Text Mining”[Title/Abstract] OR "Supervised Machine Learning"[Mesh] OR "Supervised Learning”[Title/Abstract] OR “Semi-supervised Learning”[Title/Abstract] OR “Semi supervised Learning”[Title/Abstract] OR “Learning from Labeled Data”[Title/Abstract] OR "Unsupervised Machine Learning"[Mesh] OR "Unsupervised Learning”[Title/Abstract] OR "Neural Networks, Computer"[Mesh] OR “artificial neural network”[Title/Abstract] OR “Neural Network”[Title/Abstract] OR “Natural Language Processing"[Mesh] OR “Natural Language Processing”[Title/Abstract] OR “chat-gpt”[Title/Abstract] OR “chatgpt”[Title/Abstract] OR “gpt”[Title/Abstract]) AND (“Nursing” [Mesh] OR “Nursing”[Title/Abstract] OR “Critical Care Nursing” [Mesh] OR “Critical Care Nursing ”[Title/Abstract] OR “Intensive Care Nursing”[Title/Abstract] OR “Emergency Nursing” [Mesh] OR “Emergency Nursing”[Title/Abstract] OR “Emergency Room Nursing”[Title/Abstract] OR “Perioperative Nursing” [Mesh] OR “Perioperative Nursing” [Title/Abstract] OR “Operating Room Nursing” [Title/Abstract] OR “Postanesthesia Nursing” [Title/Abstract] OR “Post-Anesthesia Nursing” [Title/Abstract] OR “Post Anesthesia Nursing” [Title/Abstract] OR “Post Surgical Nursing” [Title/Abstract] OR “Post-Surgical Nursing” [Title/Abstract] OR “Recovery Room Nursing” [Title/Abstract] OR “Trauma Nursing” [Mesh] OR “Trauma Nursing” [Title/Abstract] OR “Nurses” [Mesh] OR “Nurse” [Title/Abstract])

Supplementary Table S3. Quality appraisal of studies included.

Quantitative descriptive studies	Approach	MMAT criteria for quantitative descriptive studies				
		Is the sampling strategy relevant to address the research question?	Is the sample representative of the target population?	Are the measurements appropriate?	Is the risk of nonresponse bias low?	Is the statistical analysis appropriate to answer the research question?
Meyfroidt et al., 2011	Observational	Y	Y	Y	U	Y
Mutegeki et al., 2023	Observational	U	Y	Y	Y	Y
Nazzal et al., 2020	Observational	Y	Y	Y	Y	Y
De Koning et al., 2023	Retrospective	Y	Y	Y	Y	Y
Zaboli et al., 2024	Observational	U	Y	Y	N	Y
Gu et al., 2024	Observational	Y	Y	Y	Y	Y
Horgn et al., 2017	Observational	Y	Y	Y	U	Y
Ivanov et al., 2021	Retrospective	Y	Y	Y	Y	Y
Cramer et al., 2019	Observational	Y	Y	U	Y	Y
Yu et al., 2020	Retrospective	Y	Y	U	Y	Y
Tsumoto et al., 2006	Observational	U	Y	U	U	Y
Lee et al., 2018	Observational	U	Y	Y	U	Y
Wang et al., 2023	Retrospective	Y	Y	Y	Y	Y
King et al., 2023	Retrospective	Y	Y	Y	Y	Y
Chang et Chang, 2019	Observational	Y	Y	U	Y	Y
Bhattacharyya et al., 2022	Observational	Y	Y	Y	Y	Y
Xu et al., 2022	Retrospective	Y	Y	Y	Y	Y
Ladios-Martin et al., 2020	Retrospective	Y	Y	Y	U	Y
Toledo et al., 2024	Observational	U	Y	Y	U	Y
Cui et Jin, 2023	Observational	U	Y	Y	Y	Y
Brandao-de-Resende et al., 2023	Retrospective	Y	Y	U	Y	Y
Cho et al., 2022	Prospective	U	Y	U	U	Y
Qualitative studies	Approach	MMAT criteria for qualitative studies				
		Is the qualitative approach appropriate to answer the research question?	Are the qualitative data collection methods adequate to address the research question?	Are the findings adequately derived from the data?	Is the interpretation of results sufficiently substantiated by data?	Is there coherence between qualitative data sources, collection, analysis and interpretation?
Abraham et al. 2023	Interview	Y	Y	Y	Y	Y
Mixed-methods studies	Approach	MMAT criteria for mixed-methods studies				
		Is there an adequate rationale for using a	Are the different components of the	Are the outputs of the integration of	Are divergences and inconsistencies	Do the different components of the

		mixed method design to address the research question?	study effectively integrated to answer the research question?	qualitative and quantitative components adequately interpreted?	between quantitative and qualitative results adequately addressed?	study adhere to the quality criteria of each tradition of the methods involved?
Greenbaum et al., 2019	Retrospective cohort before-and-after study, qualitative study	U	N	Y	Y	Y

Supplementary Table S4. Studies in which each performance measure is present.

Performance's measures	Number of studies
Sensitivity	15
AUC-ROC	14
Specificity	10
Precision	10
Accuracy	7
Positive predictive value	7
Negative predictive value	6
Brier score	3
Root mean squades relative error	2
Processing time (min)	2
Predicted risk	1
Hosmer-Lemeshow U-statistic	1
Loss penalty function	1
F-beta score	1
Unweighted Cohen's kappa	1
Under-triage	1
Over-triage	1
Completeness	1
Overall quality	1
Mean keystrokes required	1
Number of events	1
Calibration plot	1
AUPRC	1
R square	1
Mean absolute error	1
Intraclass correlation coefficient	1

AUC- ROC = Receiver Operating Characteristics Area under the Curve; AUPRC = area under precision recall curve.

Supplementary Table S5. Distribution and combinations of input and output data types in AI models.

	**OUTPUT**					
**INPUT**	Continuous	Categorical	Binary	Text	Binary/Text	Total
Continuous/binary/categorical; unstructured data		2	1			3
Continuous/categorical; unstructured data	2	5				7
Continuous/binary; unstructured data		1				1
Continuous; unstructured data	2		1			3
Continuous/binary/categorical	2	3				5
Continuous/binary	1					1
Continuous/categorical		1				1
Unstructured data				2	1	3
Total	7	12	2	2	1	24
